# Maggot Extract Interrupts Bacterial Biofilm Formation and Maturation in Combination with Antibiotics by Reducing the Expression of Virulence Genes

**DOI:** 10.3390/life12020237

**Published:** 2022-02-04

**Authors:** Mustafa Becerikli, Christoph Wallner, Mehran Dadras, Johannes M. Wagner, Stephanie Dittfeld, Birger Jettkant, Falk Gestmann, Heinz Mehlhorn, Tim Mehlhorn-Diehl, Marcus Lehnhardt, Björn Behr

**Affiliations:** 1 Department of Plastic and Reconstructive Surgery, BG University Hospital Bergmannsheil, Ruhr-University Bochum, 44789 Bochum, Germany; mustafa.becerikli@rub.de (M.B.); c.wallner88@gmail.com (C.W.); mdadras@outlook.com (M.D.); max.jay.wagner@googlemail.com (J.M.W.); dittfeld.stephanie@gmail.com (S.D.); marcus.lehnhardt@bergmannsheil.de (M.L.); 2 Department of General and Trauma Surgery, BG University Hospital Bergmannsheil, Ruhr-University Bochum, 44789 Bochum, Germany; jettkant@ipa-dguv.de; 3 Alpha-Biocare GmbH, 41468 Neuss, Germany; gestmann@alphabiocare.de (F.G.); mehlhorn@uni-duesseldorf.de (H.M.); tim.mehlhorn@alphabiocare.de (T.M.-D.)

**Keywords:** maggot extract, *Lucilia sericata*, biofilm, virulence genes, *Pseudomonas aeruginosa*, *Staphylococcus aureus*, antibiotic resistance

## Abstract

Biofilms are aggregates of bacteria encased in an extracellular polymer matrix that acts as a diffusion barrier protecting the microbial community. Bacterial communication occurs by small signaling molecules called quorum sensing (QS) factors, which are involved in the activation of virulence genes and formation of biofilms. Larvae of the green bottle blowfly *Lucilia sericata* remove necrotic tissue by mechanical action (debridement) and proteolytic digestion. We produced a freeze-dried storable powder from larval extract and investigated its therapeutic effect on biofilms. Larval extract in concentrations of 6 and 12 mg/mL in combination with 0.5% antibiotics (≙50 U/mL penicillin and 50 μg/mL streptomycin) diminished free-floating (planktonic) *Pseudomonas aeruginosa* maintenance, while it showed no effect on S*taphylococcus aureus* and was not toxic to dermal cells. We established an ex vivo human dermal wound model. Larval extract in concentrations of 24 and 75 mg/mL in the presence of antibiotics (0.5%) significantly destroyed the biofilm stability of both *P. aeruginosa* and *S. aureus* biofilms. Furthermore, SEM analyses revealed crack and gap formations on *P. aeruginosa.* biofilm surface and decreased expression of *P. aeruginosa* biofilm maturation and virulence genes (*lasR*, *rhlR* and *rhlA*) was observed after treatment by larval extract in combination with antibiotics.

## 1. Introduction

Bacterial biofilms are complex structures consisting of microbial communities encased in extracellular polymer matrix containing polysaccharides, proteins, and nucleic acids [1,2]. Although antibiotics still show great therapeutic effects on individual, free-floating (planktonic) bacteria, their effect on biofilms is inherently negligible [3]. One mechanism of biofilm resistance to antibiotic agents is that the biofilm layer acts as a diffusion barrier reducing the rate of antibiotic penetration into the full depth of biofilms [4,5]. Even the long-term use of antibiotics in high concentrations does not guarantee the elimination of microorganisms within biofilms [6]. A further mechanism is that extracellular matrix protects chronic wound biofilms from the inflammatory processes of the host, which plays a significant role in wound healing [7,8]. Mature biofilms can persist in humans for many months or even over the lifetime of the host [9]. Biofilm formations on chronic wounds constitute a major clinical problem given the provision of ideal environmental conditions for biofilm aggregation. Attachment of bacteria is supported by necrotic tissue and debris, while infection of wounds is accelerated by impaired host immune response [10,11]. After the attachment of planktonic bacteria, micro-colonies consisting of primary colonizers rapidly develop and begin to produce the extracellular matrix and to express the biofilm-phenotype [12]. Furthermore, bacteria within the micro-colony begin to release quorum sensing (QS) factors, small molecular weight signaling molecules. QS is a bacterial density dependent form of cell–cell communication, involving a hierarchy of signaling molecules. It plays a key role in the activation of virulence genes and development, formation and maturation of biofilms. QS molecules have been identified in many bacterial species, and QS mutants are not able to form structurally normal biofilms [12,13].

The Gram-negative bacteria *Pseudomonas aeruginosa* (*P. aeruginosa*) and Gram-positive bacteria *Staphylococcus aureus* (*S. aureus*) are mostly responsible for delayed healing and infection in both acute and chronic wounds and are widely known to cause chronic biofilm-based infections in their hosts [14,15]. QS mechanisms of *P. aeruginosa* and *S. aureus* have been extensively studied. The initial *P. aeruginosa* QS phase is regulated by two tightly controlled pathways, namely Las (*lasR*/*lasI* genes) and Rhl (*rhlR*/*rhlI* genes) systems [16]. Analyses of 40 clinical isolates of *P. aeruginosa* revealed that 100% of the isolates expressed the *lasR* and *rhlR* genes [17]. The Las system is at the top of the QS cascade. The *lasR* gene codes the transcription factor, which is responsible for the activation of numerous target genes related to QS in *P. aeruginosa*, amongst other things *rhlR* (and further downstream factors like *rhlA*), which encode the second QS pathway [17,18].

Maggot debridement therapy is an alternative medication option of infected chronic non-healing wounds and was introduced into clinical practice by Baer in 1931 [19]. It is widely accepted that larvae of the green bottle blowfly *Lucilia sericata* (*L. sericata*) remove necrotic tissue by mechanical action (debridement) and by proteolytic digestion [20]. However, some points are disadvantageous in classic maggot debridement therapy: (1) pain during the maggot movements on the wound, (2) an annoying smell of maggot excrements, (3) logistical problems, (4) the larvae are freshly inserted and must be replaced after a few days and (5) a general feeling of disgust in the patient. To avoid these negative features, freeze-dried extracts were produced by homogenization and extraction from maggots to obtain it as a storable powder.

In this study, the effect of *L. sericata* larval extract (trade name Larveel^®^) in combination with antibiotics was investigated on biofilm formation and maturation and antibiotic resistance of *P. aeruginosa* and *S. aureus*. For this approach, in vitro co-culture experiments with human dermal cells were performed and an ex vivo human dermal wound model was used. Furthermore, biofilm surface analysis by scanning electron microscopy and measurement of gene expression of virulence factors was performed with and without Larveel^®^ treatment. Larval extract in the presence of antibiotics significantly destroyed biofilm stability of *P. aeruginosa* and *S. aureus* and significantly decreased expression of *P. aeruginosa* biofilm maturation and virulence genes (*lasR*, *rhlR* and *rhlA*).

## 2. Materials and Methods

### 2.1. Lucilia Sericata Larvae Extract

Larveel^®^, a sterile powder made from 100% larvae of *L. sericata* (Figure S1), was produced by Alpha-Biocare GmbH (Neuss, Germany) by its own patented process (patent: DE 102005061246 A1). In short, third-instar larvae of *L. sericata* grown under GMP conditions were homogenized, heated, sterile filtered, freeze-dried and sterilized with gamma radiation. Larveel^®^ powder of one vial (0.15 g) was reconstituted in 2 mL sterile DMEM (according to the tested conditions with or without antibiotics) to generate 100% Larveel^®^ stock solution (≙ 75 mg/mL). Further dilutions were made by diluting the stock solution with medium or Luria–Bertani (LB) broth (Carl Roth GmbH, Karlsruhe, Germany).

### 2.2. Bacteria Cultures

Gram-negative bacterial strain *P. aeruginosa* PAO1 (*P. aeruginosa*) and Gram-positive bacterial strain *S. aureus* 6850 (*S. aureus*), consequently expressing green fluorescent protein (gfp) and red fluorescent protein (rfp), respectively, were kindly provided by Prof. Dr. Katharina Riedel (Institute of Microbiology, University of Greifswald, Greifswald, Germany). Bacteria were inoculated into LB broth and incubated overnight at 37 °C. A total of 1 mL of overnight culture was transferred to 5 mL fresh LB broth for 4 hours. Cultures were centrifuged and bacterial pellets were dissolved in phosphate-buffered saline (PBS, Gibco, Thermo Fisher Scientific, Waltham, MA, USA) or LB broth and were diluted to OD_600_ = 0.5.

### 2.3. Isolation of Human Cutaneous Cells

Isolation of human fibroblasts and keratinocytes was performed as previously described and with some modifications [21,22]. Skin explants were obtained from adult healthy patients undergoing abdominoplasty surgery. Briefly, skin was washed several times in PBS and subcutaneous fat was excised. Subsequently, skin was completely covered with freshly prepared 0.2% dispase solution (4.7 units/mL, Gibco). After overnight incubation at 4 °C, epidermis was detached and incubated in a trypsin/EDTA solution (0.05%/0.02%, PAN-Biotech GmbH, Aidenbach, Germany). Harvested keratinocytes were cultivated in keratinocyte growth medium (Keratinocyte Growth Medium 2, PromoCell, Heidelberg, Germany) at 37 °C with 5% CO_2_. The dermis was completely covered with collagenase type-2 solution (3000 units/mL, Gibco) and incubated overnight at 37 °C. Cell suspension was filtered through a cell strainer, centrifuged and harvested fibroblasts were cultured in DMEM (Gibco), containing 10% FBS (Gibco) and 1% antibiotics (≙ 100 U/mL penicillin and 100 μg/mL streptomycin, PAN-Biotech GmbH), here entitled fibroblast medium, at 37 °C with 5% CO_2_. Media were changed every second day.

### 2.4. Cell Co-Culture with Bacteria In Vitro

Isolated fibroblasts (2 × 10^4^ cells) and keratinocytes (3 × 10^4^ cells) were seeded in confluent co-culture in 500 µL of co-culture medium (1:1 Keratinocyte Growth Medium and fibroblast medium, with 0.5% as the final antibiotics concentration) into wells of 24-well plates and cultivated overnight. This allows a rudimentary and fast co-cultivation model of fibroblasts and keratinocytes over a few days. The two antibiotics penicillin and streptomycin were used because they are predominantly applied in cell culture, are non-toxic to the examined dermal cells and are effective against both used bacterial strains. On the next day, the co-culture medium was replaced by a fresh medium and inoculated with 5 µL of *P. aeruginosa* (OD_600_ = 0.5) or 10 µL of *S. aureus* (OD_600_ = 0.5) or both bacterial strains. Larveel^®^ was added in different concentrations (0, 3, 6 and 12 mg/mL). After a 24-hour incubation, nuclei were counterstained with the fluorescence DNA dye DAPI and co-cultures were analyzed by an inverse Olympus X83 microscope (*P. aeruginosa* green fluorescence, *S. aureus* red fluorescence). Afterwards, co-cultures were fixed in 4% paraformaldehyde (Sigma-Aldrich, St. Louis, MO, USA) overnight at 4 °C and Gram staining was performed according to manufacturer’s instructions. Pictures of dermal cells (fibroblasts and keratinocytes) were taken and quantification was performed using the software Photoshop (Adobe Systems, San Jose, CA, USA). Three random regions of interest were selected (1000 × 1000 Px) and non-colonized area was semi-automatically quantified by unstained pixels via magic wand tool (tolerance 40%; noncontiguous).

### 2.5. Skin Tissue Culture, Epidermal Wounding and Infection of Ex Vivo Dermal Model

Skin tissue culture was performed as previously described and with some modifications [23]. Skin explants were obtained from adult healthy patients undergoing abdominoplasty surgery. Briefly, skin was washed several times in phosphate buffered saline (PBS) and subcutaneous fat was excised. Tissue was sliced into rectangular pieces of about 1 × 1 cm and placed into 12-well tissue culture inserts (Sarstedt, Nümbrecht, Germany) with the epithelial-side upward. Inserts were transferred to 12-well cell culture plates filled with 1.2 mL of medium DMEM (Gibco), supplemented with 10% fetal bovine serum (FBS) (Thermo Fisher Scientific), 1% antibiotics (penicillin (100 U/mL) and streptomycin (100 μg/mL), PAN-Biotech GmbH), and Amphotericin B (2.5 µg/mL, PAN-Biotech GmbH) for antimycotic medication. Samples were cultured at the air–liquid interphase at 37 °C in a humidified atmosphere containing 5% CO_2_ for 24 h. Accordingly, culture medium was changed with DMEM containing 10% FBS and 1% antibiotics.

For wounding, skin was punched standardized 1 mm in depth using a 6 mm Ø biopsy punching knife. A punched piece was removed carefully by lifting it with forceps and cutting it off with scissors, without penetrating the skin tissue. Wound infection was performed by application of 10 µL of bacterial solution (OD_600_ = 1.0) on the wound, and at the same time, addition of 10 µL of the medium containing 48 or 150 mg/mL Larveel^®^ and 1% antibiotics to obtain a final concentration of 24 or 75 mg/mL Larveel^®^ and 0.5% antibiotics. For control, 10 µL of the medium without Larveel^®^ but with antibiotics was used. Again, samples were cultured at 37 °C in a humidified atmosphere containing 5% CO_2_ for 24 h. Afterwards, to remove unstable fragments of biofilms, samples were washed in a standardized manner with defined volume (*P. aeruginosa:* 500 µL, *S. aureus:* 100 µL) of PBS application once before samples were fixed with 4% paraformaldehyde (PFA) at 4 °C overnight. Accordingly, after washing three times with PBS, samples were incubated in 10% saccharose at 4 °C overnight before preparing frozen sections. Sections were Gram stained according to standard protocols. Pictures were taken and the number of pixels of biofilms was quantified using the software Photoshop as described. The biofilm area without Larveel^®^ treatment was defined as 100%. All experiments were repeated seven times. Results of all experiments were given as mean ± SD.

### 2.6. Scanning Electron Microscopy (SEM)

SEM was performed as previously described [24] with some modification. A total of 50 µL of LB broth with *P. aeruginosa* (OD_600_ = 1.0) was added to 50 µL of LB broth supplemented with the appropriate final concentrations of Larveel^®^, 0.2% antibiotics, and a final glucose concentration of 0.25% as a carbon source on small titanium specimens that were placed into wells of 6-well plates and were incubated for 48 h at 37 °C. Bacteria were fixed in 3% glutaraldehyde at 4 °C overnight, followed by dehydration using an ethanol series. After sticking onto metal stubs and gold/palladium (60%/40%) coating in a sputter coater (Emitech K500×, Gala Instruments, Bad Schwalbach, Germany), specimens were analyzed by SEM (DSM 962, Zeiss, Oberkochen, Germany).

### 2.7. RNA Preparation and cDNA Synthesis

RNA isolation and cDNA synthesis was performed as previously described [25] with some modification. Briefly, 50 µL of LB broth with *P. aeruginosa* (OD_600_ = 0.5) was added to 50 µL of LB broth supplemented with 0.25% glucose in a final concentration in 96-well plates at 37 °C. Depending on the experimental conditions, antibiotics (0.25% final concentration) or Larveel^®^ (24 mg/mL final concentration) were added. After 48 h, RNA was isolated separately from planktonic bacteria in LB supernatant and biofilms attached on plate surfaces by an RNAprotect Bacteria Reagent and RNeasy Protect Bacteria Kit (Qiagen, Hilden, Germany) according to manufacturer’s instructions. Purified RNA was eluted in 30 µL of RNase-free water. For reverse transcription, 1 µg total RNA per reaction was transcribed using the High-Capacity cDNA Reverse Transcription Kit with RNase inhibitor (Thermo Fisher Scientific) following the manufacturer’s instructions.

### 2.8. Quantitative Real-Time PCR (qRT-PCR)

Quantitative relative gene expression was determined on Applied Biosystems StepOnePlus real-time PCR System using SYBR Green master mix (Thermo Fisher Scientific) and primers (TIB Molbiol, Berlin, Germany) listed in Table S1 [26]. For quantitative analysis, 2 μL of cDNA, 2 µL H_2_O, 1 µL of primer mix (0.5 µM each sense and antisense) and 5 µL of SYBR Green Mastermix in a total volume of 10 μL were used for each reaction. The cycling conditions were as follows: 95°C for 10 min, 40 cycles consisting of 95 °C for 5 s, annealing 60 °C for 30 s, 72 °C for 10 s; followed by a melting point analysis: 65–95 °C at a ramp speed of 0.5 °C/s; and finally, a cooling phase. Data were analyzed according to the manufacturer’s ΔΔC_t_ method (Applied Biosystems, Thermo Fisher Scientific, Waltham, MA, USA). Samples were normalized to the *oprF* gene. Results are shown relatively in relation to gene expression without Larveel^®^ and antibiotic treatment, that was defined as 1.0.

### 2.9. Statistical Analysis

All experiments were repeated at least three times. Results of all experiments were given as mean ± SD. Results without Larveel^®^ treatment were defined as 100% in all relative results. Statistical analyses were performed by unpaired 2-tailed Student’s *t*-test. *p*-values < 0.05 were considered statistically significant and indicated in the figures as follows: *: *p* < 0.05; **: *p* < 0.01; and ***: *p* < 0.001.

## 3. Results

### 3.1. Cell Protective Effect of Larveel^®^ towards Bacterial Infection in Combination with Antibiotics

**Figure 1:**
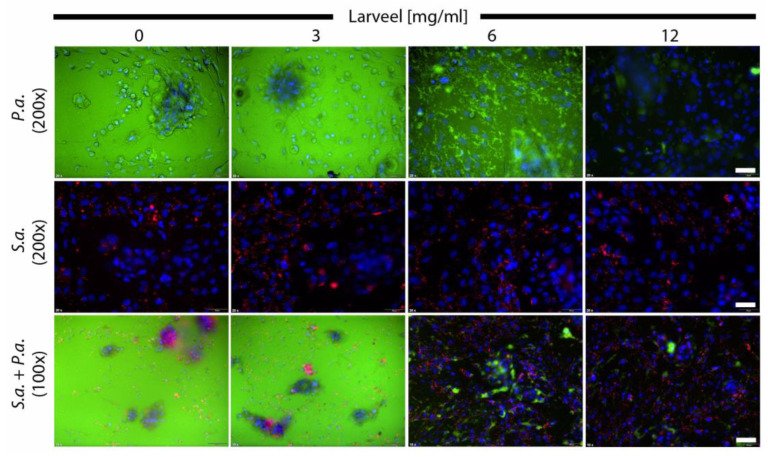
Fluorescence microscopy of cells infected with bacteria. Fibroblast and keratinocyte co-cultures were infected with *P. aeruginosa* and *S. aureus* in medium with 0.5% antibiotics and were supplemented with different Larveel^®^ concentrations for 24 h. *P. aeruginosa* is shown in green fluorescence (gfp), *S. aureus* in red fluorescence (rfp) and nuclei of dermal cells are shown in blue (DAPI). Nuclei of keratinocytes were arranged in clusters. P.a.: *P. aeruginosa*; S.a.: *S. aureus*. Top and middle line 200× magnification, scale bar: 50 µm; bottom line 100× magnification, scale bar: 100 µm.

To analyze the effect of Larveel^®^ on cells infected with Gram-negative and Gram-positive bacteria, fibroblast and keratinocyte co-cultures were infected with *P. aeruginosa* or *S. aureus* or both strains. Additionally, medium was supplemented with 0.5% antibiotics and different concentrations of Larveel^®^. Dermal cells inoculated with *P. aeruginosa* showed strong green fluorescence indicating massive bacterial growth in the medium (Figure 1, top). Furthermore, diffuse and destroyed fibroblast and keratinocyte cell nuclei (in blue) were observed in these wells. Addition of Larveel^®^ in concentrations of 6 and 12 mg/mL diminished green *P. aeruginosa* fluorescence in a concentration-dependent manner. In contrast, *S. aureus* was observed as red fluorescent dots (Figure 1, middle), thus a vigorous bacterial proliferation was not observed with 0.5% antibiotics. No striking differences in *S. aureus* distribution and red fluorescence intensity after Larveel^®^ application were seen in this experimental approach. *P. aeruginosa* and *S. aureus* co-culture with dermal cells exhibited an overgrowth of *P. aeruginosa* in the medium (Figure 1, bottom). Similar to the top line, the addition of Larveel^®^ in concentrations of 6 and 12 mg/mL diminished the green *P. aeruginosa* fluorescence. Co-culture approaches with bacteria and with 0% antibiotics exhibited completely cloudy media due to severe bacterial growth and were discarded (not shown). Thus, we decided to use 0.5% antibiotics, on the one hand to allow the bacteria to grow, but on the other hand, to prevent uncontrolled expansion. A basal concentration of 0.5% antibiotics was constantly necessary in co-culture experiments to keep the eukaryotic cells alive and maintain the experimental conditions.

**Figure 2:**
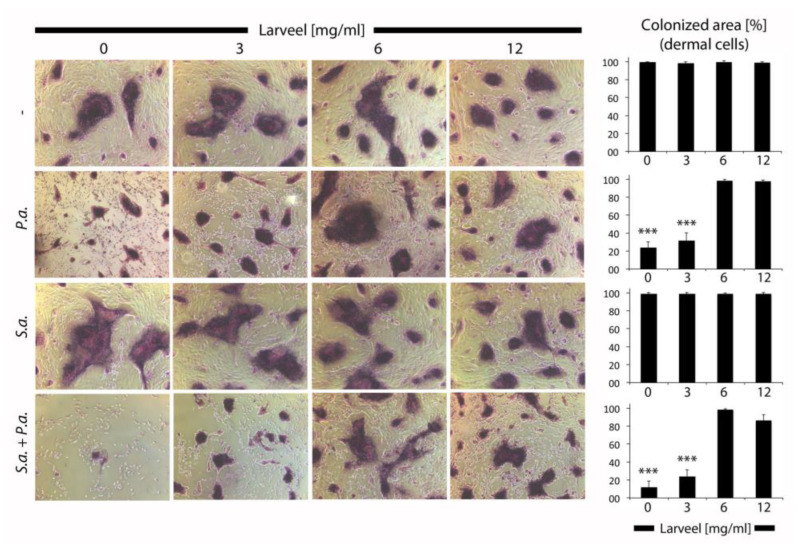
Gram staining of cells infected with bacteria. Fibroblast and keratinocyte co-cultures infected with *P. aeruginosa* and *S. aureus* in medium with 0.5% antibiotics and different Larveel^®^ concentrations were stained 24 h after incubation. Pictures of dermal cells were taken (50× magnification) and number of pixels was quantified. P.a.: *P. aeruginosa*; S.a.: *S. aureus*. Colonized area by dermal cells without bacterial inoculation and Larveel^®^ treatment was defined as 100%. Results of all experiments were given as mean ± SD. *** *p* < 0.001.

Analyses of dermal cells after infection with bacteria by Gram staining revealed a toxic effect of *P. aeruginosa* on fibroblasts and keratinocytes, and thus destroyed cell structures were recognized (Figure 2). *S. aureus* showed no observable effect on dermal cells. In summary, Larveel^®^ concentrations of 6 and 12 mg/mL in combination with 0.5% antibiotics showed no toxic effect on dermal cells, rather protective effect towards bacterial infections.

### 3.2. Generation of an Ex Vivo Human Dermal Wound Model

**Figure 3:**
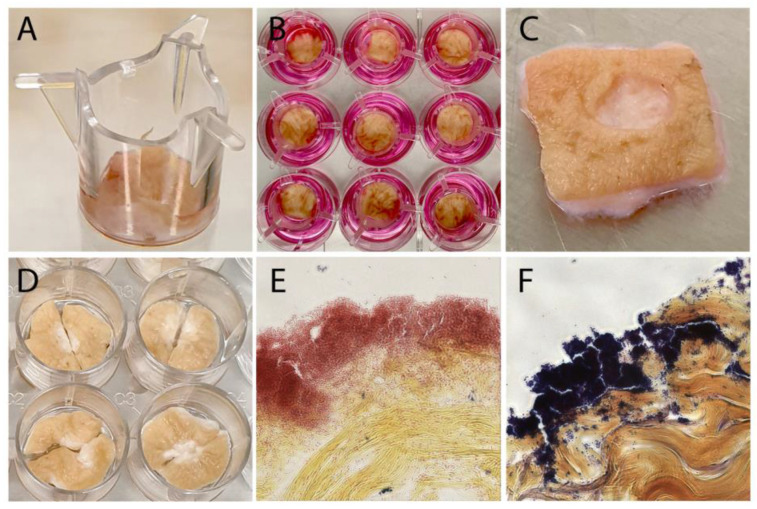
Ex vivo human dermal wound model. (**A**–**D**) Dermal tissue pieces were cultivated in 12-well tissue culture plates in air–liquid interphase and were standardized biopsy punched. Gram staining of (**E**) *P. aeruginosa* and (**F**) *S. aureus* biofilms on infected skin wounds (400× magnification).

In order to analyze Larveel^®^ treatment depending biofilm stability and for quantification of the effect of Larveel^®^ on biofilm removal, an ex vivo human dermal wound model was established. Initially, ex vivo experiments were carried out as previously described [23] and were later modified. Human dermal tissue pieces cultivated in 12-well tissue culture plates in air–liquid interphase were biopsy punched in a standardized manner and infected for 24 h (Figure 3). Gram staining revealed reddish Gram-negative *P. aeruginosa* and deep purple Gram-positive *S. aureus* biofilms on the skin tissue (Figure 3E,F).

### 3.3. Impaired Biofilm Maturation and Stability by Larveel^®^ Treatment in the Ex Vivo Human Dermal Wound Model

**Figure 4:**
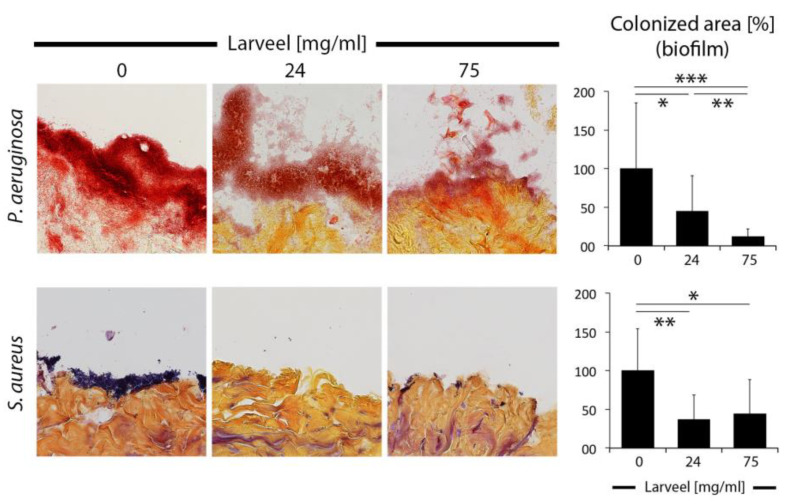
Destruction and removal of biofilms by Larveel^®^ treatment in the ex vivo human dermal wound model. Skin wounds were infected and treated at the same time with Larveel^®^ and 0.5% antibiotics for 24 h. After PBS washing one-time by standardized manner, samples were fixed and Gram stained. Pictures were taken (200× magnification) and number of pixels of biofilms was quantified. Biofilm area without Larveel^®^ treatment was defined as 100%. Results of all experiments were given as mean ± SD. * *p* < 0.05, ** *p* < 0.01, *** *p* < 0.001.

After establishment of an experimental dermal model with clinical relevance, we performed Larveel^®^ treatment with two therapeutic concentrations and examined biofilm maturation and stability. For this approach, based on the routine wound care, in which infected wounds are cleaned, samples were standardized washed with defined volume, distance and speed of PBS application one-time before fixation and staining. *P. aeruginosa* produced thick and robust biofilms, which also infiltrated the infected tissue. Untreated biofilms remained unchanged after washing. Treatment with 24 and 75 mg/mL Larveel^®^ solution (in presence of 0.5% antibiotics) significantly destroyed biofilm stability depending on Larveel^®^ concentration and flaky biofilm structures were observed (Figure 4). Compared to *P. aeruginosa* biofilms, *S. aureus* biofilms were more superficial and slighter. Treatment with both Larveel^®^ concentrations significantly removed remaining biofilms and only small bacterial spots were observed (Figure 4).

### 3.4. Crack Formation in P. aeruginosa Biofilm Surface after Larveel^®^ Treatment in SEM

**Figure 5:**
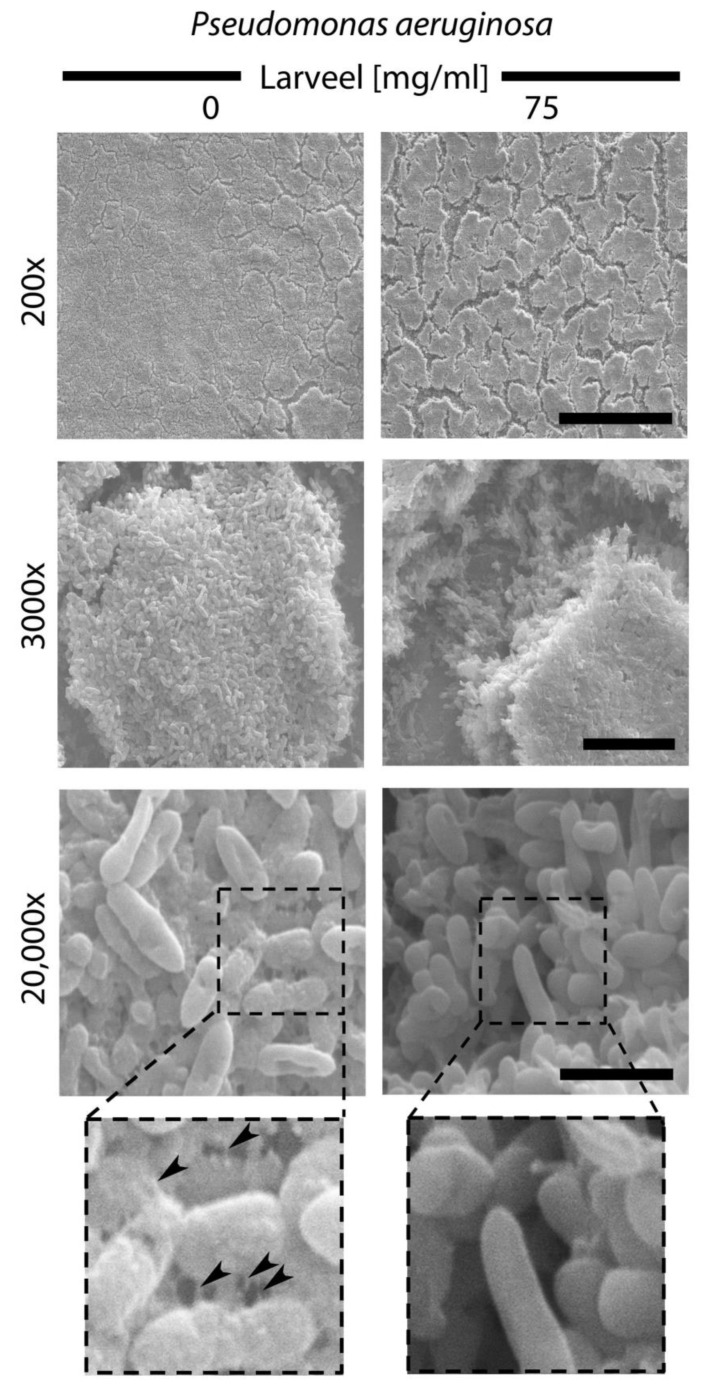
SEM analyses of *P. aeruginosa* biofilm surface. Biofilm production and maturation was performed for 48 h with and without Larveel^®^ treatment. Cracks were observable on the surface of biofilms of treated *P. aeruginosa* (**top**). Filament-like extracellular matrix structures on the surface of untreated bacteria are indicated by arrowheads (**down**; box: enlarged sections). Scale bars: 200×: 200 µm; 3000×: 10 µm; 20,000×: 2 µm.

To find clues regarding why biofilms were easier removed after Larveel^®^ treatment, we performed SEM analyses of *P. aeruginosa* biofilms. Compared to untreated samples, Larveel^®^ addition resulted in cracks and formation of gaps in *P. aeruginosa* biofilm surface (200×, 3000× magnification). At higher resolution (20,000×), filament-like extracellular matrix structures that seem to connect bacterial cells with each other and with their environment were observed. In contrast, the surface of single bacteria in the Larveel^®^ treated group appeared to be smoother. Extracellular matrix structures observed on bacterial surface disappeared after Larveel^®^ treatment (Figure 5).

### 3.5. Decreased Expression of Biofilm Maturation and Virulence Genes after Larveel^®^ Treatment

**Figure 6:**
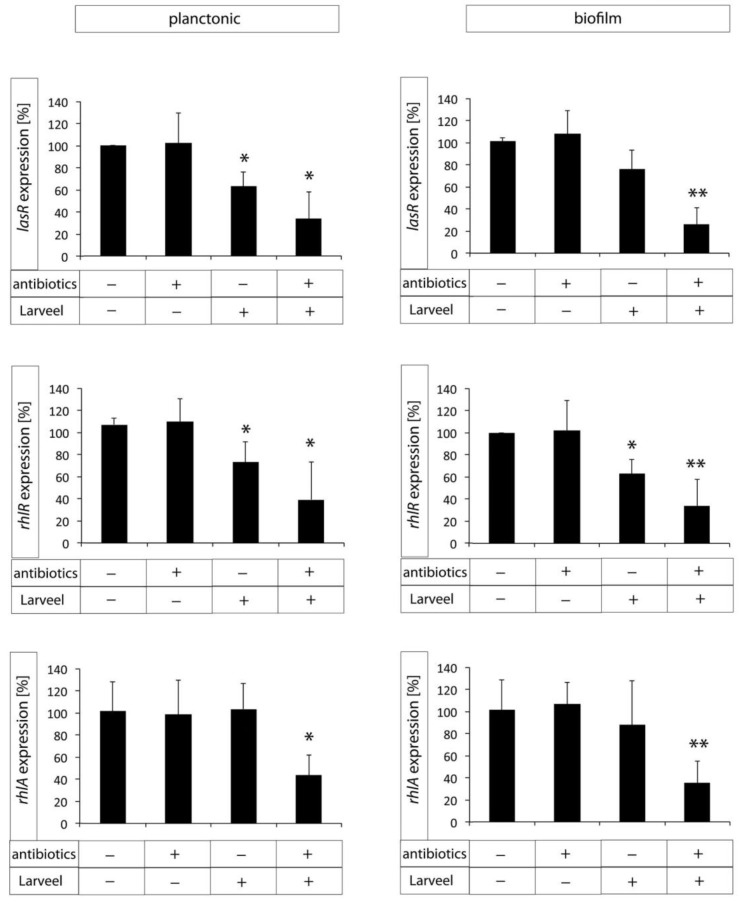
Gene expression of biofilm maturation and virulence genes measured by qRT-PCR after treatment with antibiotics (0.25%) or/and Larveel^®^ (24 mg/mL). Gene expression without antibiotics and Larveel^®^ was defined as 100%. Results of all experiments were given as mean ± SD. (* *p* < 0.05, ** *p* < 0.01).

RNA was isolated separately from planktonic bacteria in LB supernatant and biofilms attached on plate surfaces. Interestingly, supernatant with planktonic bacteria was more viscous in untreated controls, whereas after Larveel^®^ treatment supernatant was more fluid (data not shown). To investigate both pathways of *P. aeruginosa* QS initial phase, gene expression of one factor of each pathway (*lasR* and *rhlR*) were quantified by qRT-PCR. Additionally, gene expression of *rhlR* downstream factor *rhlA* was measured. Both antibiotics used in this study (penicillin and streptomycin) showed no effect on gene expression of the analyzed QS-associated genes when they were used alone without Larveel^®^. Gene expression of QS activation factor *lasR* was significantly decreased after a combination of antibiotics and Larveel^®^ in planktonic bacteria as well as in biofilms (Figure 6). Additionally, Larveel^®^ alone without antibiotics significantly inhibited *lasR* expression in planktonic bacteria. Similarly, gene expression of *rhlR* was also significantly diminished after combination therapy, but addition of Larveel^®^ alone without antibiotics also decreased *rhlR* gene expression in both planktonic bacteria and biofilms. Gene expression of *rhlA* was not affected by antibiotics or Larveel^®^ solely. Only the combination of both resulted in significantly decreased *rhlA* gene expression in planktonic bacteria as well as in biofilms (Figure 6).

## 4. Discussion

The steady increase in antibiotic resistance of bacteria present on wounds is a major problem that will become more dramatic in the near future. Extensive use of antibiotics creates an evolutionary stress response in the bacterial population that over time leads to a rapid evolution of resistant strains. Thus, the amount of antibiotics used must be drastically reduced to avoid further antimicrobial resistance.

Larvae/maggots are natural products and maggot debridement therapy could support the wound treatment without the use of antibiotics in high concentrations and severe side effects. The majority of studies concerning the effect of maggots on eukaryotic cells or bacteria revealed no influence on dermal cell viability, proliferation, migration and angiogenesis or no direct antibacterial effect in vitro. However, in clinical observations maggot therapy proved to be successful [27,28]. We also observed no toxic effect of Larveel^®^ on dermal cells. Furthermore, a cell protective effect of Larveel^®^ towards bacterial infection in combination with antibiotics was determined.

Larveel^®^ is already being used successfully in clinical applications in some developing countries at a concentration of 75 mg/mL without seeing any toxic effects on the patient ([29] and clinical observation). It is generally known that a therapeutic in vitro concentration is not enough in an in vivo experiment. In in vivo experiments, significantly higher dosages are required. Based on this fact and our previous experiments, we opted for reducing the concentration in our in vitro experimental setup and we opted for lower concentrations and reduced it to 3–12 mg/mL. Because the ex vivo setup is close to clinical in vivo usage, we decided to increase the Larveel^®^ concentration according to the clinical concentration. Furthermore, in order to clearly show the effect on the biofilms, in SEM examinations we decided to use the usual clinical concentration of 75 mg/mL.

Even planktonic bacteria in suspensions are mechanically coupled to each other, this coupling gets intensified in biofilms [30]. Biofilms survive despite antibiotic therapy or the innate and adaptive immune system of the patient. Consequently, many biofilm infections are difficult to treat efficiently and the demand for research of new treatment strategies is of particular clinical relevance. One problem at this point is that biofilms protect bacterial infections against antibiotic treatment regiments. To overcome this, biofilms have to be made permeable. We observed significantly more crack and gap formation in *P. aeruginosa* biofilm surfaces after Larveel^®^ treatment and less extracellular matrix structures on higher resolution. In previous studies, it was observed that short-chain fatty acids inhibited biofilm formation [31]. Utilizing gas chromatography–mass spectrometry (GC–MS) analysis (data not shown), we detected isovaleric acid (3-methylbutanoic acid), a short-chain fatty acid, is contained in Larveel^®^. Furthermore, it is assumed that Larveel^®^ contains various D-amino acids due to the fly maggots used [32,33]. Some D-amino acids exhibited synergistic anti-biofilm activity in combination with antibiotics [34]. Thus, treatment with Larveel^®^ in the presence of antibiotics significantly interrupted biofilm formation and maturation by a synergistic effect, but Larveel^®^ alone could not achieve this result.

*P. aeruginosa* and *S. aureus* are one the most common cause of cutaneous infections [35,36]. Experiments with *P. aeruginosa* and *S. aureus* revealed that both bacterial strains could coexist initially, but in late-stage co-cultures, *P. aeruginosa* predominates in the community and eliminates *S. aureus* [37,38]. To avoid this predomination in our experimental period, we started the experiments with more *S. aureus*. Moreover, in the present study, we focused on QS of *P. aeruginosa*. QS is a bacterial cell-to-cell communication that involves a hierarchy of signaling molecules, which in pathogenic bacteria is associated with virulence regulation and biofilm formation [39]. It allows bacteria to synchronously adjust behavior in response to changes in the population density and species composition of the environment community [40,41]. Furthermore, QS enables bacteria to communicate with other species as well [42]. Considering that more than 10% of *P. aeruginosa* genes are regulated by QS, the critical role of QS in physiological adaptation is more comprehensible [43]. These genes are mainly involved in virulence factor production, motility-sessility switch and biofilm development and antibiotic resistance mechanisms [43].

Analogously, mechanisms interfering with bacterial cell-cell communication evolved, which was termed quorum quenching [42]. A variety of small-molecule inhibitors of QS signaling were discovered and were tested as novel antimicrobial therapies [44,45,46,47]. However, there are still typical major obstacles in the transition from the bench to the clinic, such as lack of potency in animal models, toxicity, stability and delivery [48]. Furthermore, some antibiotics, such as azithromycin and ceftazidime or chemical compounds, were observed to decrease the expression of QS-regulated virulence factors in *P. aeruginosa* [49,50,51]. In our approach, we determined decreased expression of biofilm maturation and virulence genes in *P. aeruginosa* after Larveel^®^ treatment. To our knowledge, this is the first-time that reduced gene expression of QS factors by maggot larvae extract is described.

Larveel^®^ is designed to be applied in saline solution directly onto the wounded or rather infected tissue before it is covered with a dressing. To reduce the time required to change wound dressings and to simplify the procedure, sterile and storable wound cover containing Larveel^®^ has been developed (Figure S2). The sheets passed all necessary quality criteria and initial studies on patients with chronic wounds are in progress.

In summary, this study demonstrates that larval extract of the green bottle blowfly *Lucilia sericata* interrupts biofilm formation and maturation of *P. aeruginosa* and *S. aureus* in synergistic combination with antibiotics. No toxic effect of Larveel^®^ on dermal cells was observed. Larveel^®^ application resulted in crack formation in *P. aeruginosa* biofilm surface and reduction of extracellular matrix structures on bacterial surfaces. Finally, a significant decrease of gene expression of virulence genes was detected.

## 5. Conclusions

Bacterial biofilms on chronic wounds constitute a serious health burden. From a clinical point of view, efficient treatment of chronic wounds attacked by these complex structures belongs to one of the most difficult complications. Despite little effects of antibiotics on biofilms, increasing resistance to antibiotics is one of the main global concerns at present. Thus, any approach that can help to tackle this task should be taken into consideration. In this study, we demonstrated that Larveel^®^, an extract of the green bottle blowfly *Lucilia sericata*, destroys biofilm stability of *P. aeruginosa* and *S. aureus* in combination with antibiotics. Furthermore, *P. aeruginosa.* biofilms reveal crack formation on surface and decreased expression of virulence genes after therapy. In this context, we showed that local treatment of bacterial biofilms on chronic wounds with Larveel^®^ in combination with antibiotics might be considered a good candidate for successful therapy of biofilms.

## Data Availability

The data that supports the findings of this study are available in the figures, tables and the Supplementary Materials of this article.

## Supplementary Materials

The following are available online at https://www.mdpi.com/article/10.3390/life12020237/s1, Figure S1: Larveel^®^, a sterile powder made from 100% larvae of *Lucilia sericata*, Figure S2: Larveel^®^ wound dressing assembled with content of one vial per sheet; Top: front side for wound contact assembled with Larveel^®^; Down: hydrophobic blue backside, Table S1: Primer sequences (5′→3′) for qRT-PCR.
