# Supplementary material for: Maggot Extract Interrupts Bacterial Biofilm Formation and Maturation in Combination with Antibiotics by Reducing the Expression of Virulence Genes
Source: Life (Basel). 2022 Feb 4;12(2):237. doi: 10.3390/life12020237 (PMC8875949; doi:10.3390/life12020237)
Supplement: Supplementary file 1 [file life-12-00237-s001.zip › life-1551784-supplementary.pdf]

life

MDPI

Article

Maggot Extract Interrupts Bacterial Biofilm Formation and Maturation in Combination with Antibiotics by Reducing the Expression of Virulence Genes

Mustafa Becerikli [1], Christoph Wallner [1], Mehran Dadras [1], Johannes M. Wagner [1], Stephanie Dittfeld [1], Birger Jettkant [2], Falk Gestmann [3], Heinz Mehlhorn [3], Tim Mehlhorn-Diehl [3], Marcus Lehnhardt [1] and Björn Behr [1]*

[1] Department of Plastic and Reconstructive Surgery, BG University Hospital Bergmannsheil, Ruhr-University Bochum, 44789 Bochum, Germany; mustafa.becerikli@rub.de (M.B.); c.wallner88@gmail.com (C.W.); mdadras@outlook.com (M.D.); max.jay.wagner@googlemail.com (J.M.W.); dittfeld.stephanie@gmail.com (S.D.); marcus.lehnhardt@bergmannsheil.de (M.L.)

[2] Department of General and Trauma Surgery, BG University Hospital Bergmannsheil, Ruhr-University Bochum, Bochum, Germany; jettkant@ipa-dguv.de

[3] Alpha-Biocare GmbH, Neuss, Germany; gestmann@alphabiocare.de (F.G.); mehlhorn@uni-duesseldorf.de (H.M.); tim.mehlhorn@alphabiocare.de (T.M.-D.)

* Correspondence: bjorn.behr@rub.de; Tel.: +49-234-302-3443

Citation: Becerikli, M.; Wallner, C.; Dadras, M.; Wagner, J.M.; Dittfeld, S.; Jettkant, B.; Gestmann, F.; Mehlhorn, H.; Mehlhorn-Diehl, T.; Lehnhardt, M.; et al. Maggot Extract Interrupts Bacterial Biofilm Formation and Maturation in Combination with Antibiotics by Reducing the Expression of Virulence Genes. *Life* **2022**, *12*, 237. https://doi.org/10.3390/life12020237

Academic Editor: Cheng-Yen Kao

Received: 28 December 2021
Accepted: 2 February 2022
Published: 4 February 2022

Publisher's Note: MDPI stays neutral with regard to jurisdictional claims in published maps and institutional affiliations.

Copyright: © 2022 by the authors. Licensee MDPI, Basel, Switzerland. This article is an open access article distributed under the terms and conditions of the Creative Commons Attribution (CC BY) license (http://creativecommons.org/licenses/by/4.0/).

Table S1. Primer sequences (5′→3′) for qRT-PCR.

Primer name	Sequence
lasR F	ACGCTCAAGTGGAAAATTGG
lasR R	GTAGATGGACGGTTCCCAGA
rhlR F	AGGAATGACGGAGGCTTTTT
rhlR R	CCCGTAGTTCTGCATCTGGT
rhlA F	CGAGGTCAATCACCTGGTCT
rhlA R	GACGGTCTCGTTGAGCAGAT
oprF F	GGTTACTTCCTGACCGACGA
oprF R	TCGCTGTTGATGTTGGTGAT

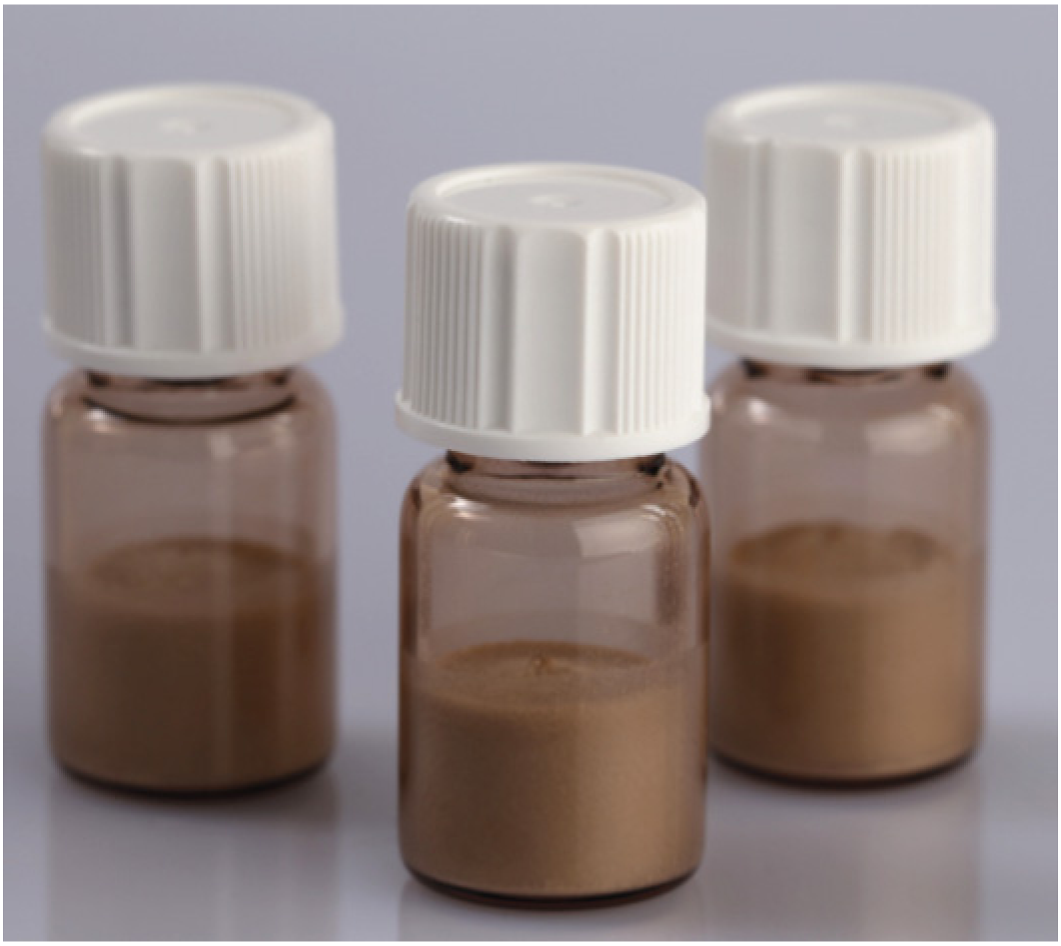

Figure S1. Larveel®, a sterile powder made from 100% larvae of *Lucilia sericata.*

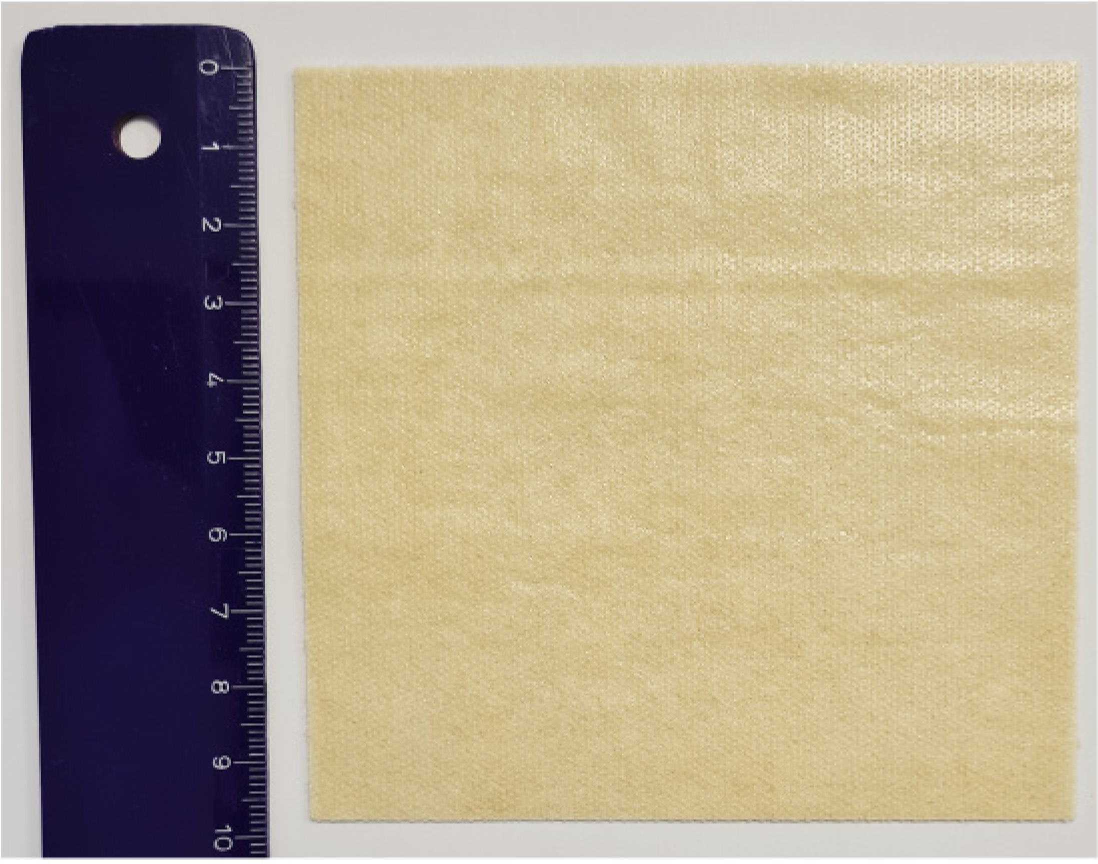

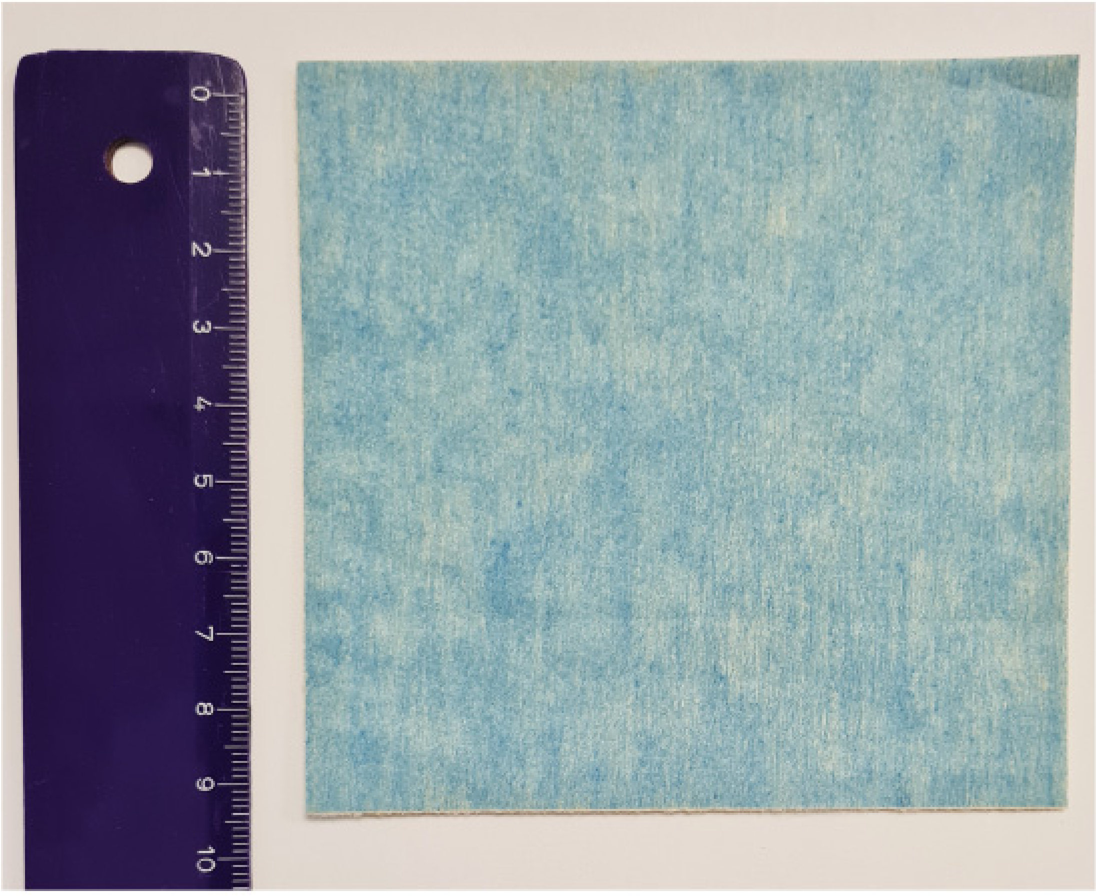

Figure S2. Larveel® wound dressing assembled with content of one vial per sheet; Top: front side for wound contact assembled with Larveel®; Down: hydrophobic blue backside.
